# Clinical and histopathological study of 279 dentigerous cysts in 192 dogs (2012–2022)

**DOI:** 10.3389/fvets.2024.1412089

**Published:** 2024-05-23

**Authors:** Senni Vesterinen, Laura Lönnberg, Jouni J. T. Junnila, Niina Luotonen, Helena Kuntsi

**Affiliations:** ^1^Anident Veterinary Clinic, Kirkkonummi, Finland; ^2^EstiMates Oy, Espoo, Finland

**Keywords:** dentigerous cyst, unerupted teeth, dog, histopathology, odontogenic cyst, cysts, retrospective study

## Abstract

Unerupted teeth in dogs are fairly common and may develop an odontogenic cyst that causes destruction of the surrounding bone and affect adjacent teeth. We analyzed histological reports of cysts associated with unerupted teeth in a large population of dogs. Medical records and histopathological results of cysts associated with unerupted teeth were evaluated from all dogs treated at a private referral veterinary dental clinic over a 10-year period (2012–2022). A total of 192 dogs with 279 cysts associated with one or more unerupted teeth were included in the study. Brachycephalic breeds were overrepresented. The most affected were Tibetan Spaniels with 58 dogs (30%) and Boxers with 48 dogs (25%). The most common affected tooth was the mandibular first premolar tooth with 238 (84%) cysts. Of the total of 279 cysts, 208 (75%) were histopathologically examined. None of the cysts examined contained malignant changes. Based on these 208 cysts, the probability of finding a cyst with malignant changes in a population of dogs is 0–1.4% (confidence interval 95%).

## 1 Introduction

A cyst is defined as an epithelial-lined pathologic cavity (1). Odontogenic cysts are lined with odontogenic epithelium and occur in the tooth-bearing parts of the maxilla and mandible (1). Literature reports the following types of canine odontogenic cysts: dentigerous cyst, lateral periodontal cyst, gingival cyst, periapical (radicular) cyst, furcation cyst, keratinized odontogenic cyst, and canine odontogenic parakeratinized cyst (1–4). The most common type is the dentigerous cyst (1). Of all histopathologically examined oral lesions in dogs, odontogenic cysts account for 9% (5).

**Figure 1:**
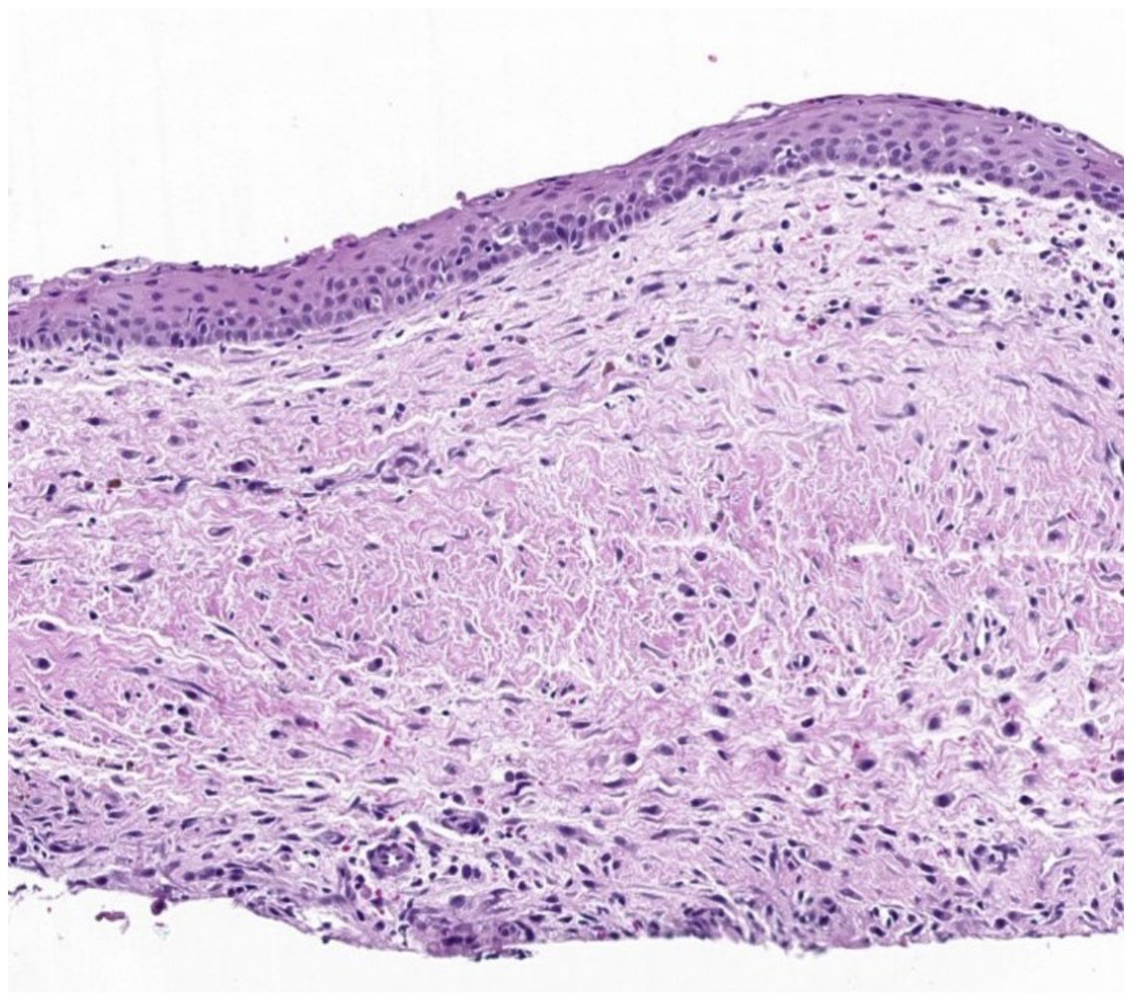
Histological image of a wall of a typical dentigerous cyst. The wall consists of fibrovascular tissue lined by non-keratinized stratified squamous epithelium, with occasional inflammatory cells, hemorrhage, and hemosiderin.

The odontogenic cyst wall consists of a thin layer of non-keratinized or para-keratinized stratified squamous epithelium (Figure 1). The lumen may consist of hemorrhagic or serous fluid, a small number of leukocytes, and degraded cellular debris. Secondary inflammation is a common finding in all types of odontogenic cysts. Histological features are not specific to any types of odontogenic cysts, apart from keratin in keratinized odontogenic cysts (1, 3, 4, 6). The presence of ameloblasts is suggestive of an odontogenic tumor, as they should not be present in any types of odontogenic cysts (1).

Unerupted teeth can either be embedded or impacted. An embedded tooth is unerupted because of a lack of eruptive force, whereas an impacted tooth is covered with a physical barrier blocking the eruption path (7). An unerupted tooth carries a risk of developing a dentigerous cyst which, by definition, is an epithelial-lined and fluid-filled cavity around the crown of an unerupted tooth. In a normally erupted tooth, the enamel organ epithelium atrophies and merges with the overlying mucosal epithelium to form the initial crevicular epithelium of the newly erupted tooth (8). When a tooth fails to erupt, the alteration of the enamel organ epithelium may lead to accumulation of fluid between the enamel organ epithelium and the crown of the unerupted tooth (9). The increasing fluid volume causes destruction of the surrounding bone by bone resorption factors, may affect the adjacent teeth and, in severe cases, even lead to a mandibular fracture or invade the nasal cavity (7, 9).

Histopathologic diagnosis of a dentigerous cyst requires visualization of the attachment of the cyst wall to the cementoenamel border of an unerupted tooth. In the majority of histological preparations made from biopsy specimens, this may be not possible, and the histopathological diagnosis therefore is an odontogenic cyst without further specification of a dentigerous cyst. For the clarity of this paper, we use the term dentigerous cyst for all cysts associated with unerupted teeth.

**Figure 2:**
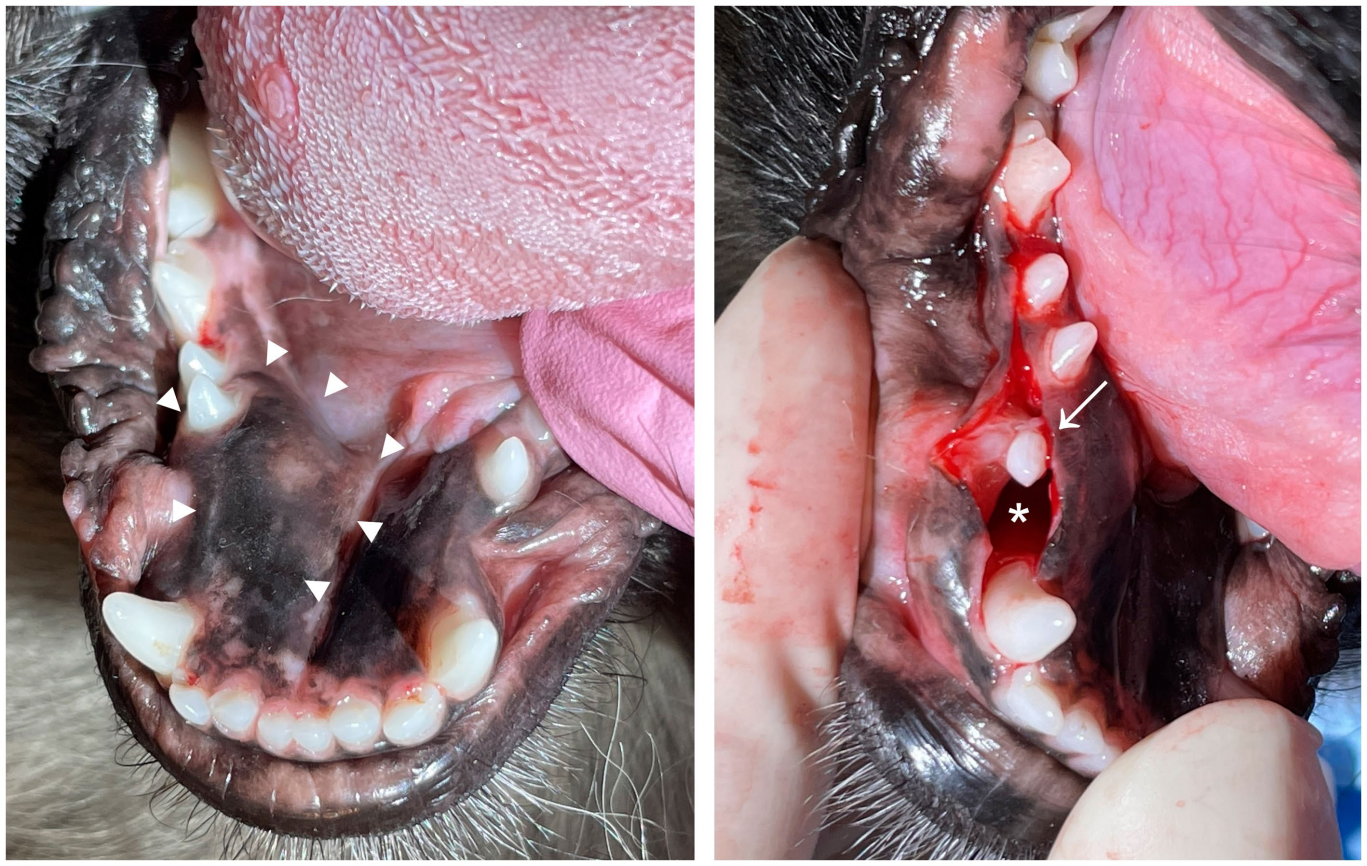
Three-year-old Tibetan spaniel with a dentigerous cyst associated with an unerupted right mandibular first premolar tooth. **(A)** Before operation. Note the slight swelling in the area (arrowheads). **(B)** Intraoperative photograph of the unerupted right mandibular first premolar tooth (arrow) and cyst cavity (asterisk).

**Figure 3:**
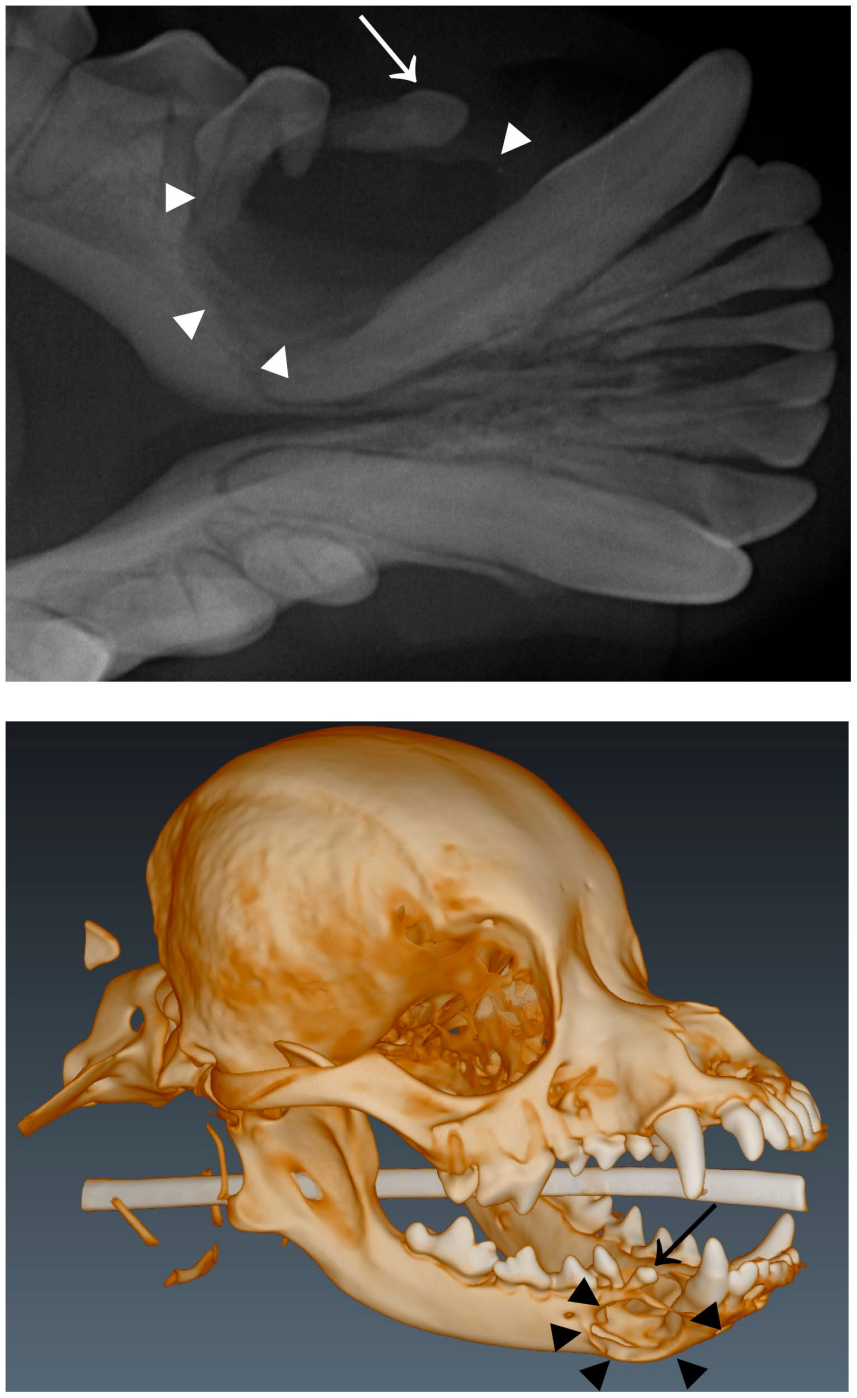
**(A)** Dental radiograph of the same dog as in Figure 2 with a dentigerous cyst (arrowheads) associated with an unerupted right mandibular first premolar tooth (arrow). **(B)** 3D-construction image from cone-beam computed tomography of the same dog with a cyst (arrowheads) associated with an unerupted right mandibular first premolar tooth (arrow).

In dogs, an unerupted tooth causes a dentigerous cyst in 29–49% cases (6, 10, 11). The most frequently affected teeth are the mandibular first premolar and canine teeth (4, 11). Clinically, a dentigerous cyst appears as a visibly absent tooth and, in some cases, intraoral swelling in the area (4) (Figure 2). There is typically no mucosal inflammation or erosion. A typical radiological finding is a unilocular, circular radiolucency with a well-defined cortex that encloses the crown of an unerupted tooth (12) (Figure 3). The treatment includes extraction of the unerupted tooth, careful enucleation of the cyst lining and, in severe cases, a bone graft (7). This treatment has proved to be successful, with no recurrence of the cysts (11). Marsupialization with later extirpation has also been described as an effective treatment method (13).

Published veterinary literature raises some concerns about odontogenic cysts with evidence of histopathologically malignant changes (14, 15). However, in these cases, no cysts with malignant changes were associated with unerupted teeth. Several reports regarding cysts associated with unerupted teeth describe other histopathological diagnoses apart from dentigerous cysts (or those presenting concurrently with an odontogenic cyst). They include canine acanthomatous ameloblastoma, osteosarcoma, compound odontoma, ameloblastic odontoma, ameloblastic fibro-odontoma, invasive squamous papilloma, cystic peripheral odontogenic fibroma, and squamous cell carcinoma (10, 11, 16–20). To our knowledge, there are no reports in the veterinary literature that confirm the transition of a dentigerous cyst to malignant tissue.

The purpose of this study was to analyze histopathological reports of cystic lesions associated with unerupted teeth in a larger number of dogs than has previously been reported. We hypothesized that malignant changes in cysts associated with unerupted teeth are extremely rare.

## 2 Materials and methods

The data were collected retrospectively from the medical records of a private veterinary dentistry referral clinic in Finland. We included records of all client-owned dogs with a diagnosis of a cyst associated with an unerupted tooth in the 2012–2022 period. The diagnosis was based on clinical findings and radiography and/or cone-beam computed tomography and was made by or under the supervision of a board-certified veterinary dentist. The medical records were searched for all unerupted teeth, but only the ones associated with a cyst were included. The following keywords were used: unerupted, cyst, dentigerous cyst, DTC (Dentigerous cyst), T/U (Unerupted tooth), T/I (Impacted tooth), enucleation, bone graft, and osteoallograft. Exclusion criteria included dogs with cysts not associated with an unerupted tooth and dogs with an unerupted tooth but without a cyst.

The following data were obtained: breed, sex, age, unerupted tooth with a cyst, and histopathological diagnosis. For dogs that had at least one dentigerous cyst, information about other unerupted teeth without a cyst was collected. Histopathological diagnoses were made by two separate private veterinary pathology services (Rest Associates, United Kingdom and Solumo, Finland).

Based on their breed, the dogs were classified as either brachycephalic or non-brachycephalic. For this variable, mixed breed dogs were excluded. The following breeds were classified as brachycephalic: Boston Terrier, Boxer, Chihuahua Long-haired, Chihuahua Smooth-haired, Chow Chow, French Bulldog, Japanese Chin, Pug, and Tibetan Spaniel (21–23). The following breeds were classified as non-brachycephalic: American Staffordshire Terrier, American Toy Fox Terrier, Basenji, Belgian Malinois, Border Collie, Border Terrier, Coton de Tuléar, Finnish Spitz, German Shorthaired Pointer, Giant Schnauzer, Golden Retriever, Hovawart, Karelian Bear Dog, Labrador Retriever, Landseer, Miniature Poodle, Miniature Schnauzer, Nova Scotia Duck Tolling Retriever, Pembroke Welsh Corgi, Pomeranian, Russian Toy, Schapendoes, Spanish Water Dog, and Toy Poodle (23).

The total number of dogs treated was calculated for each breed using the clinic’s medical data. The data provided from the Finnish Kennel Club database were used for the total number of dogs registered during the study period in each breed. As the Finnish Kennel Club does not register mixed breed dogs, they were excluded from the analysis of this variable.

Frequency tables and descriptive statistics were calculated for the study variables (sex, age, breed, brachycephalic and non-brachycephalic dogs, and number of unerupted teeth without a cyst) using the number of individual dogs as the divisor. In addition, teeth with a cyst and the number of cysts were summarized using the total number of cysts as the divisor.

The probability of finding a cyst with a sign of histopathologically malignant changes in a population of dogs treated at the clinic was evaluated by calculating the 95% confidence interval utilizing the Wilson score method.

The proportions of individual dogs with a cyst associated with unerupted teeth of all patients treated at the clinic were calculated in total by breed and by brachycephalic/non-brachycephalic conformation. Similarly, the proportion of all dogs treated at the clinic to all registered dogs in the Finnish Kennel Club was calculated in total, by breed and by brachycephalic/non-brachycephalic conformation.

All statistical calculations were performed using SAS software version 9.4 (SAS Institute Inc., Cary, NC, US).

## 3 Results

A total of 192 dogs with 279 dentigerous cyst were included in the study. Ninety-four dogs (49.0%) were males (76 intact and 18 neutered) and 98 (51.0%) females (76 intact and 22 neutered). The mean and median ages for the dogs at the time of treatment were 4.5 and 4.1 years, respectively, ranging from 6 months to 12 years.

The study population consisted of 34 different breeds, the most common being the Tibetan Spaniel (58 dogs, 30.2%) and Boxer (48 dogs 25.0%) (Table 1). Brachycephalic dogs had 222 (79.6%) of 279 cysts. Tibetan Spaniels and Boxers alone had 170 (60.9%) of all cysts.

**Table 1:** Study population.

Breed	N of dogs with cysts	N of dogs treated at the clinic	% of dogs with cyst treated at the clinic	N of dogs registered	Proportion of dogs treated at the clinic to registered dogs	Proportion of dogs with cyst to registered dogs
American Staffordshire Terrier	2	49	4.08%	2,189	2.24%	0.09%
American Toy Fox Terrier	2	13	15.38%	92	14.13%	2.17%
Basenji	1	15	6.67%	748	2.01%	0.13%
Belgian Malinois	1	158	0.63%	2059	7.67%	0.05%
Border Collie	1	161	0.62%	5,987	2.69%	0.02%
Border Terrier	2	88	2.27%	2,581	3.41%	0.08%
Boston Terrier	3	40	7.50%	2,296	1.67%	0.13%
Boxer	48	88	54.55%	2,401	3.67%	2.00%
Chihuahua Long-haired	2	113	1.77%	6,200	1.82%	0.03%
Chihuahua Smooth-haired	14	218	6.42%	6,724	3.24%	0.21%
Chow Chow	1	6	16.67%	1,210	0.50%	0.08%
Coton de Tuléar	3	67	4.48%	6,050	1.11%	0.05%
Finnish Spitz	1	16	6.25%	7,376	0.22%	0.01%
French Bulldog	2	45	4.44%	4,833	0.93%	0.04%
German Shorthaired Pointer	1	22	4.54%	3,037	0.72%	0.03%
Giant Schnauzer	1	44	2.27%	1,364	3.23%	0.07%
Golden Retriever	2	150	1.33%	14,438	1.04%	0.01%
Hovawart	1	53	1.89%	1853	2.86%	0.05%
Japanese Chin	3	14	21.43%	830	1.69%	0.36%
Karelian Bear Dog	1	7	14.29%	7,690	0.09%	0.01%
Labrador Retriever	4	374	1.07%	24,571	1.52%	0.02%
Landseer	1	16	6.25%	952	1.68%	0.11%
Miniature Poodle	3	120	2.50%	5,341	2.25%	0.06%
Miniature Schnauzer	5	121	4.13%	9,667	1.25%	0.05%
Mixed breed	5	713	0.70%	-	-	-
Nova Scotia Duck Tolling Retriever	2	68	2.94%	3,106	2.19%	0.06%
Pembroke Welsh Corgi	3	33	9.09%	3,015	1.09%	0.10%
Pomeranian	1	31	3.22%	3,036	1.02%	0.03%
Pug	12	51	23.53%	3,142	1.62%	0.38%
Russian Toy	1	23	4.34%	1,129	2.04%	0.09%
Schapendoes	1	15	6.67%	969	1.55%	0.10%
Spanish Water Dog	3	103	2.91%	5,148	2.00%	0.06%
Tibetan Spaniel	58	105	55.24%	7,625	1.38%	0.76%
Toy Poodle	1	58	1.72%	2,219	2.61%	0.05%

**Figure 4:**
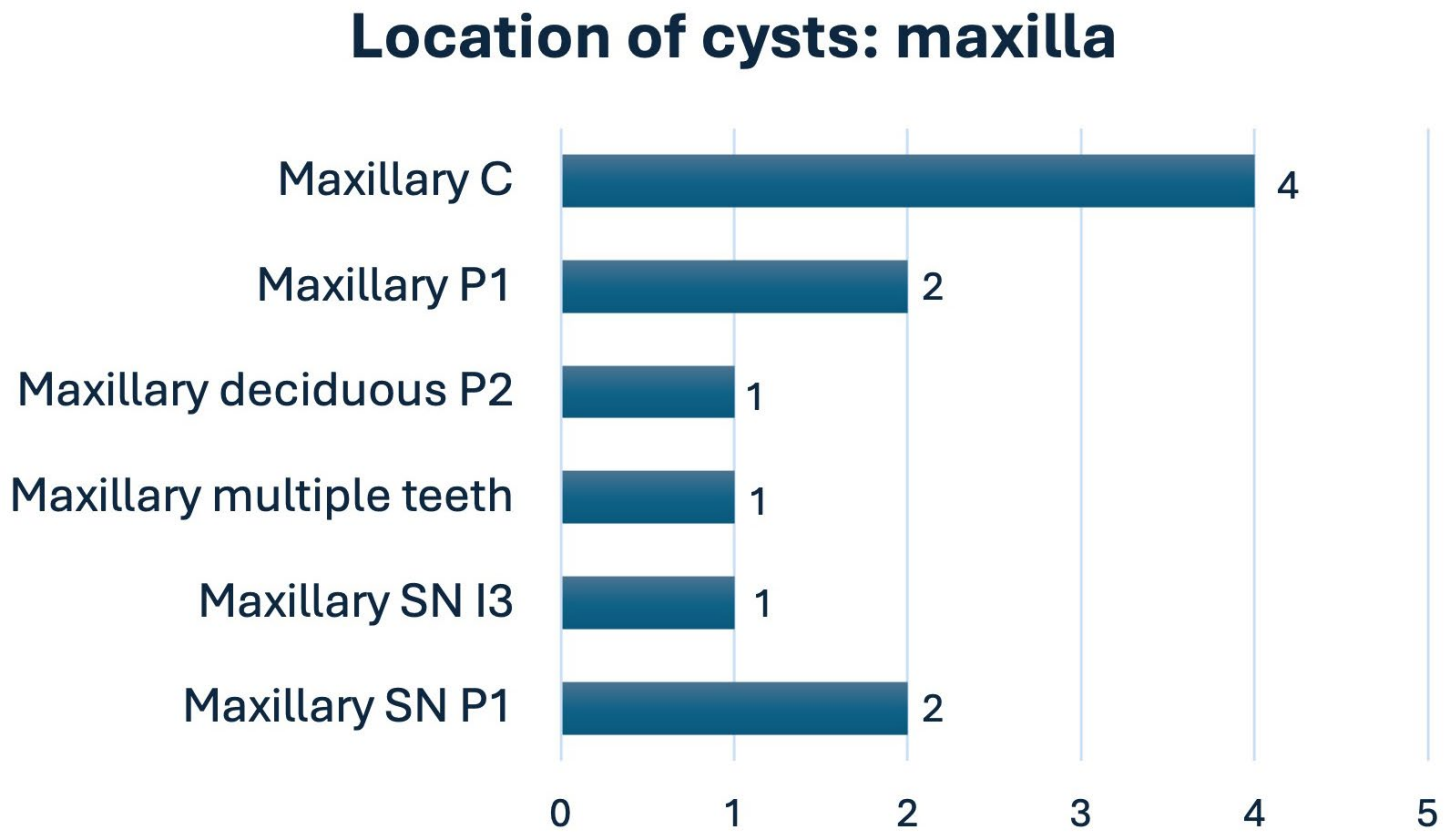
Location of cysts in the maxilla (C, canine; I, incisor; M, molar; P, premolar; SN, supernumerary).

**Figure 5:**
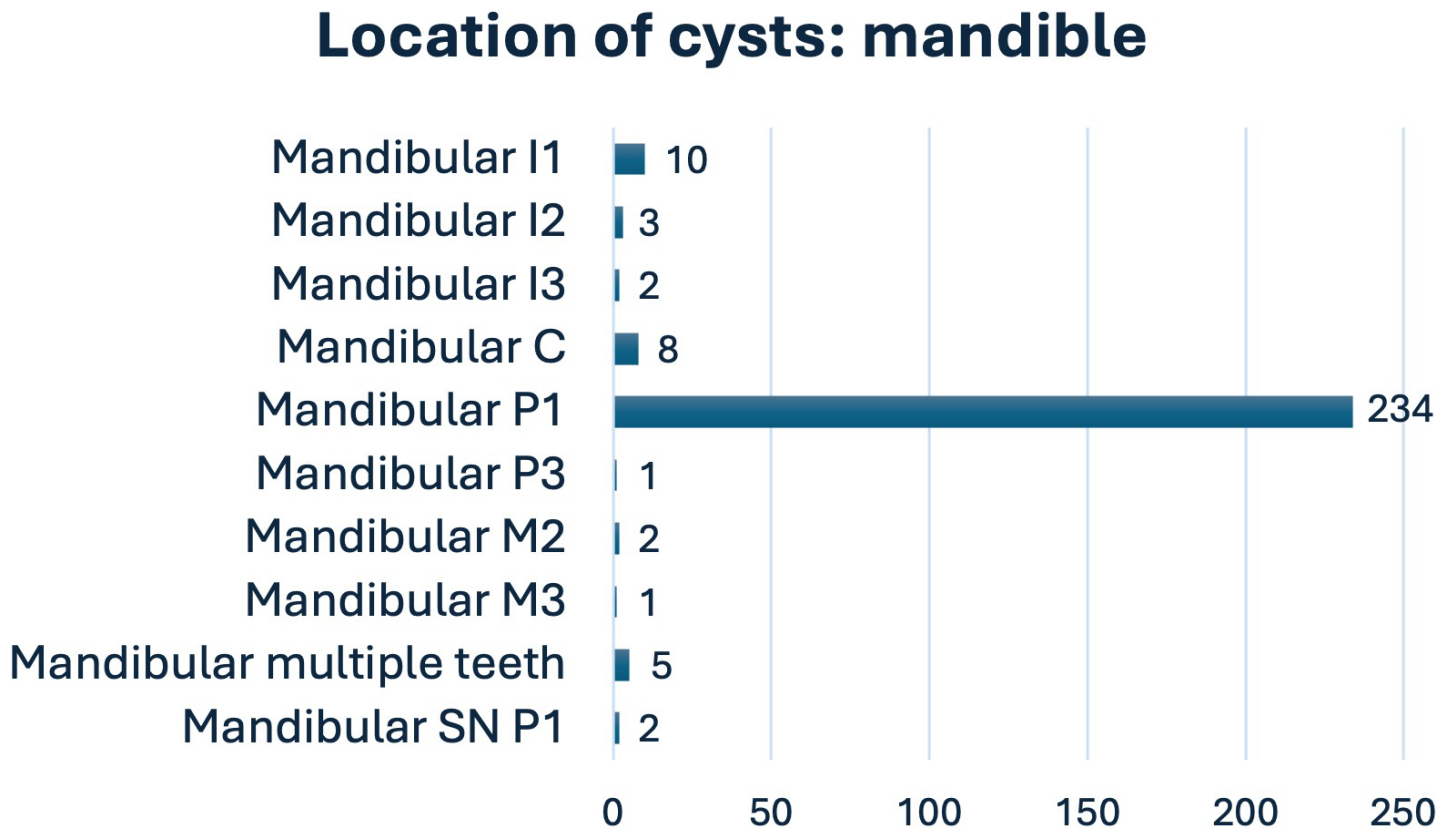
Location of cysts in the mandible (C, canine; I, incisor; M, molar; P, premolar; SN, supernumerary).

Of the 279 dentigerous cysts, most were in the mandible (96.1%). Of all cysts, 234 (83.9%) were associated with mandibular first premolar teeth and 15 (5.4%) with mandibular incisor teeth. Five (1.8%) cysts were associated with supernumerary teeth (Figures 4, 5). One cyst was associated with an unerupted deciduous maxillary second premolar tooth. In six cysts (2.2%), the dentigerous cyst was associated with multiple unerupted teeth: two with mandibular third incisor and canine teeth, one with mandibular first incisor and canine teeth, one with mandibular canine and first premolar teeth, one with mandibular first, second and third incisor teeth, and one with maxillary second and first incisor teeth.

The size of the cystic lesions varied from cysts that involved only the unerupted tooth to cysts that also affected roots of multiple adjacent teeth.

Forty-five dogs (23.4%) with at least one dentigerous cyst also had 66 unerupted teeth without clinical evidence of cyst formation. The most common tooth was mandibular first premolar tooth (39, 59.1%). The number of these teeth in one individual varied from zero to six. Thirty-six dogs (80.0%) had one unerupted tooth without a cyst. The mean age of these dogs was 4.4 years (ranging from 0.6 years to 10.8 years). There were eight dogs that were over seven years old. Brachycephalic breeds were overrepresented. The most common breeds were Boxer (13 dogs, 28.9%) and Tibetan Spaniel (11 dogs, 24.4%).

Histopathological examination was performed on 208 (74.6%) cysts. There was no evidence of malignant changes in any of the samples. In 200 cysts (71.7% of all cysts and 96.2% of examined ones), the histopathologic diagnosis was dentigerous cyst. In eight cysts (2.9% of all cysts and 3.8% of examined ones), the diagnosis of a cyst could not be established, but no evidence of neoplasia was seen. In four cysts, in which the diagnosis of a cyst could not be made, the reason was that the samples contained no epithelium. In these samples, there were other histopathologic findings that could be suggestive of a cyst. For example, in one sample, it was suspected that the hyperplastic squamous epithelium was part of the cyst lining, but there was also moderate to dense mixed inflammation. Other samples were non-diagnostic because the presence of a cyst wall could not be confirmed, and the inflammation was non-specific.

Lesions diagnosed as dentigerous cysts showed histological similarities. All samples contained at least parts of well-differentiated stratified squamous epithelium. Several lesions also included bone fragments. Secondary inflammatory findings were common. These included multifocal infiltrates of inflammatory cells (lymphocytes, plasma cells, and macrophages), hemorrhage, hemosiderosis, and cholesterol clefts.

Based on our findings, the probability of finding a cyst with malignant changes in a population of dogs treated at a veterinary dental clinic was 0–1.4% with a 95% confidence interval.

## 4 Discussion

Our study, with 279 cysts, revealed no malignant changes in cysts associated with unerupted teeth. It also confirmed the previous findings that brachycephalic breeds are predisposed to these types of cysts (10, 11). Our material comprises both the greater number of cysts and most numerous histopathologically examined odontogenic cysts than any previous study (6, 10, 11).

We found no sex predisposition for dogs with dentigerous cysts. This is in agreement with previous studies (4). In humans, dentigerous cysts are more common in males (9). The age range for dogs with cysts in our study was from 6 months to 12 years; this also concurs with other studies (6, 10, 11). Our data includes only the age of the dogs at the time when they underwent the surgery for the cyst. This does not allow us to draw conclusions about the age of the dogs at the time when the cysts started to develop. Human studies have documented that the highest incidence of dentigerous cysts occurs between 10 and 30 years of age (9).

Previous studies have pointed out that unerupted teeth and dentigerous cysts are more common in brachycephalic dog breeds (10, 11). The classification of breeds as brachycephalic varies according to different sources, as no consensus or standardized classification system to determine the skull shapes of different breeds exists (23). For example, Tibetan Spaniels and Chow Chows are classified as brachycephalic or mesocephalic, depending on the study (21–23). Brachycephalic breeds were overrepresented in our study, with 222 (80%) of the 279 cysts. The majority of them were found in Tibetan Spaniels and Boxers, which together presented with 170 (61%) of all cysts.

To find out the breed predisposition, the number of dogs of different breeds diagnosed with dentigerous cysts were compared to all dogs treated at the clinic. Since our study utilized the case material of a private veterinary dentistry referral clinic, the material is likely selectively biased. When the number of different breeds of all dogs treated at the clinic was compared to the breed registration numbers from the Finnish Kennel Club (Table 1), we were able to conclude that the proportion of dogs with cysts to registered dogs was 100 times higher for Boxers compared with Labrador Retrievers. Similarly, compared to Labrador Retrievers, in Tibetan Spaniels the proportion was 38 and in Pugs 19 times higher. The higher incidence of dentigerous cysts in brachycephalic breeds has been shown to correlate with the higher incidence of unerupted teeth in these breeds, not that in these breeds an unerupted tooth would be more likely to develop a cyst (10).

Tibetan Spaniel is an noteworthy breed because only one previous study has reported it as being predisposed to dentigerous cysts (4). In our study, Tibetan Spaniel was the most common breed with a dentigerous cyst. This may be due to the fact that Tibetan Spaniel is a common breed in Finland. In 2022, it was the fifteenth most registered breed in the Finnish Kennel Club. Another reason for the high incidence may be that the unerupted teeth are, for some reason, more common in Tibetan Spaniels in Finland than elsewhere.

Our study confirmed the previous finding that the majority (253/279, 91%) cysts are located in the mandible, with the unerupted mandibular first premolar tooth being the most prevalent tooth with cyst formation (4, 10, 11). We did not measure the size of the cysts, but according to radiographs and cone-beam computed tomography, the sizes varied from smaller cysts enclosing a single unerupted tooth to large cysts involving multiple unerupted teeth or roots of multiple adjacent teeth. Most likely, the variation of the size is due to of the interval of the diagnosis and the beginning of the cyst formation. With early diagnosis, the cysts are more likely to be small when detected. Consequently, the treatment could be easier. We agree with the recommendation that dental radiographs are taken in all cases of visibly absent teeth. It is noteworthy that in this study five cysts were associated with supernumerary teeth and one with unerupted deciduous tooth. This means that even a normal number of erupted teeth does not exclude the possibility of an unerupted tooth with a cyst.

Forty-five dogs (23% of all dogs) that presented with a dentigerous cyst also had other unerupted teeth with no clinical or radiographic evidence of cystic development. Almost all these teeth were extracted during the cyst operation, but none were sent for histopathological examination. The eight dogs older than 7 years that had both a dentigerous cyst and unerupted teeth with no cyst support the assumption that there is no individual predisposition for cyst development. Instead, the higher prevalence of unerupted teeth likely predisposes to the risk of cyst development.

Histopathological examination was not performed of 71 (25%) cysts. The reasons for not performing histopathology were the small size of the lesion, the destruction of the cyst epithelium, and the cost of the examination. Of the examined 208 samples, 200 (96%) were diagnosed as odontogenic cysts. The differentiation for a dentigerous cyst needs the visualization of the cementoenamel junction and this was not possible in many samples. A more definitive diagnosis could have been made if, in all cases, the unerupted tooth – still attached to the cyst lining - had been sent to the histopathological examination. In the eight samples where the histopathological diagnosis was not a cyst, there were four where the other histological features were strongly suggestive of a cyst but, as no epithelium was detected, the histological diagnosis of a cyst could not be made. The reasons for the absence of epithelium include small sample size, handling of the tissues, and examination technique. In the other four samples where the diagnosis of a cyst could not be made, there was no evidence of malignant or neoplastic changes.

Poulet and Summer (14) suggested that the odontogenic cyst lining may transform into an odontogenic tumor. It has also been suggested that the malignant lesions develop by themselves and only resemble odontogenic cysts at the early stages (1). The risk of tumors developing within odontogenic cysts is described in veterinary and human literature (1), but there are no veterinary reports confirming the transition of a dentigerous cyst to a malignancy. An older study (14) presented cases where tumors were suspected to have arisen from the lining of an odontogenic cyst, but since the suspected odontogenic cysts in that study were not associated with teeth, it is debatable if the initial diagnoses of odontogenic cysts were accurate. It is likely that the suspected odontogenic tumors arising from odontogenic cysts were malignant from the start and just histopathologically resembled odontogenic cysts.

Two reports of a tumor in association with an odontogenic cyst have been published. They are a canine acanthomatous ameloblastoma in association with a lateral periodontal cyst (15) and a compound odontoma in association with a dentigerous cyst (24). The case with canine acanthomatous ameloblastoma was not associated with an unerupted tooth (15). Several reports of compound odontomas with cystic lesions have been presented (17–19). In all these cases, the teeth-like structures or denticles were radiographically visible. Thus, they were radiographically distinguishable from dentigerous cysts. There are also three reports of an ameloblastic fibro-odontoma (16) and ameloblastic odontomas (10), associated with an unerupted tooth. In all cases, the patients were under 8 months old. In one of these studies, among the two ameloblastic odontomas, they also reported one invasive squamous papilloma, one cystic peripheral odontogenic fibroma, and one oronasal respiratory cyst to present with a cystic lesion associated with an unerupted tooth (10). The authors of this study did not verify whether there were any clinical or radiological features that allowed the clinician to suspect other lesions than a typical dentigerous cyst. In another study, of 50 cystic lesions associated with an unerupted tooth in dogs, two neoplastic lesions were reported: a canine acanthomatous ameloblastoma in a 5-month-old dog and an osteosarcoma in a 9-year-old dog (11). Since the clinician had recommended maxillectomy and a biopsy as a first-line procedure for these patients, it can be assumed these lesions appeared neoplastic at the initial clinical examination. In one case report squamous cell carcinoma was diagnosed in the area of an unerupted tooth. Also in this case, there were clinical signs suggestive of other pathology than a benign odontogenic cyst (20).

Limitations of this study were the retrospective nature and the selection criteria which might have biased the results in favor of benign appearing lesions.

A recommended clinical practice is to submit all removed cystic lesions for histopathological examination. Our study included 208 cysts with histopathological examination. To our knowledge, this is the largest study population of dentigerous cysts to date. No evidence of malignancy was evident in any of the samples examined. According to our data, we conclude that the prevalence of a malignant histological findings in dentigerous cysts is under 1.4% (confidence interval 95%). Previous studies with fewer cases involving cystic lesions associated with unerupted teeth also report no malignant changes in histopathological examination (6, 13). While, based on our study, malignant transformation of clinically and radiographically typical dentigerous cysts would appear to be rare, routine histopathological examination remains recommended, albeit mostly for the sake of academic completeness and good clinical practice.

## Data availability statement

The original contributions presented in the study are included in the article/supplementary material, further inquiries can be directed to the corresponding author.

## Ethics statement

Ethical review and approval were not required for the animal study because of the retrospective nature of the study. Written informed consent for participation was not obtained from the owners because this is a retrospective study where the procedures had been performed prior to the study’s conception.

## Author contributions

SV: Data curation, Investigation, Methodology, Writing – original draft, Writing – review & editing. LL: Data curation, Methodology, Writing – original draft. JJ: Data curation, Formal analysis, Writing – original draft. NL: Investigation, Writing – review & editing. HK: Conceptualization, Funding acquisition, Investigation, Methodology, Project administration, Resources, Validation, Writing – review & editing.
